# Long-term period psychiatric disorders after severe traumatic brain injuries in young male patients due to road traffic accidents

**DOI:** 10.1192/j.eurpsy.2026.11902

**Published:** 2026-07-31

**Authors:** N. Syrmos

**Affiliations:** Aristotle University, Thessaloniki, Greece

## Abstract

**Introduction:**

Introduction-According to the medical literature in the long term period after severe traumatic brain injuries often related in disabling problem such as personality changes and cognitive impairment.

**Objectives:**

Aim-Aim of this study is to evaluate 40 cases of young male patients(<45 years old). Range of age between 15-45 years old and mean age 28,5 years. Radiological investigation with MRI studies, neurological and psychiatric evaluation in all 40 cases-100%.

**Methods:**

Results-20 cases with personality changes,50%. Impaired speed and/or information processing in 10 cases and poor memory and executive problems in other 10.20 cases with personality changes, 50%, poor motivation in 8 cases and 12 cases with self-centerd tendency and less aware of the needs of others. Good rehabilitation in 30 cases,75% and moderate in 10 cases, 25%.

**Results:**

Conclusion-Patients may be: 1) lazy and thoughtless 2) disinhibited and rude 3) with agitation- aggression 4) with anxiety-depression.

**Conclusions:**

All these factors play a role in the development of various combined types of post traumatic stress disorders, such us depression, psychosis, schizophrenia and Alzheimer’s disease. Multiple combined approaches of various medical disciplines is more than necessary.

**Disclosure of Interest:**

None Declared

